# An Act of Auto‐Eroticism: Self‐Urethral Insertion of Sewing Needle by an Old Man—A Case Report With Literature Review

**DOI:** 10.1002/ccr3.9682

**Published:** 2024-12-06

**Authors:** Rao Nouman Ali, Sohaib Irfan, Wajiha Irfan, Muhammad Asif Raza, Adeel Anwaar, Riyan Imtiaz Karamat, Aymar Akilimali, Inam Ul Haq Arain

**Affiliations:** ^1^ College of Physician and Surgeons Lahore Pakistan; ^2^ Aga Khan University Hospital Karachi Pakistan; ^3^ Combined Military Hospital Multan Pakistan; ^4^ DHQ Hospital Skardu Pakistan; ^5^ Urology Suite Midcity Hospital Lahore Pakistan; ^6^ Department of Internal Medicine Rahbar Medical and Dental College Lahore Pakistan; ^7^ Department of Research, Medical Research Circle (MedReC) Goma Democratic Republic of the Congo; ^8^ Rashid Latif Medical and Dental College Lahore Pakistan

**Keywords:** case report, foreign body, needle, self‐insertion, urethra

## Abstract

Self‐insertion of foreign bodies into the urethra is uncommon and is often associated with complex psychological or behavioral factors. Many cases of foreign bodies in the urethra have been reported worldwide with variable complaints of penile pain, penile itching, dysuria, hematuria, and complete obstruction of urine. These foreign objects are managed to remove according to their size, complexity of shape, and location in the urethra. We report a case of a self‐inserted sewing needle in the urethra by an old man during an act of achieving sexual gratification, which was removed through an endoscopic approach by cystoscopy, along with a literature review. This is the fourth reported case of auto‐eroticism in which a sewing needle was used to achieve sexual pleasure, making this case report a rare one.

## Graphical abstract

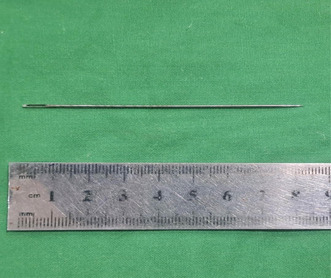

**Summary**

- Self‐insertion of foreign bodies in the urethra is rare and is often linked to complex behaviors. Symptoms include pain, itching, dysuria, hematuria, and urinary obstruction.
- We report an elderly man's case involving a sewing needle, removed via endoscopy.
- This marks the fourth documented case of auto‐eroticism using a sewing needle.

## 1 Introduction

Self‐inserted foreign bodies in the urethra are reported more frequently in men than in women. There is a wide variation in the types of objects that patients have inserted into their urethra. Previously reported items range from small pins, needles, and screws to ballpoint pens, thermometers, straws, electric wires, dining forks, metal bullets, and even larger objects such as telephone cables, nail clippers, iron rods, tongue cleaners, earphone jacks, and plastic pipes [1, 2]. There are numerous reasons for the delay in seeking medical attention, but the most common reason is that patients attempt to remove the foreign bodies themselves to avoid embarrassment [3]. These patients typically present with various complaints, including pain, swelling, anuria, and hematuria. In contrast, patients who delay treatment may develop complications such as infection, hematoma, or fistula formation. During examination, the objects are often visibly apparent due to their incomplete insertion into the urethral meatus; however, in some cases, they can only be palpated deeply within the urethra [4, 5, 6]. Investigations such as plain radiography, ultrasonography, or non‐contrast computed tomography may be required to determine the appropriate procedure for removing the objects, depending on the case. Management varies according to the location of the object within the urethra. Some cases may require only genital traction under local anesthesia, whereas the majority necessitate cystoscopic removal. In cases where cystoscopy fails, open surgical techniques are employed [7, 8, 9]. The act of self‐inserting objects is predominantly observed in patients with a history of psychiatric illness or in sexually deviant males who engage in this behavior for sexual pleasure during masturbation. This phenomenon is also reported in pediatric age groups, likely due to curiosity about body orifices. Additionally, some older patients have claimed to insert objects into their urethra as a form of self‐therapy to alleviate penile itch. This behavior is also seen in some desperate prisoners who inflict self‐harm to escape the hostile environment [10].

William J. Robinson first introduced the term “paraphilia” in 1903, which was later defined by Friedrich Salomon Krauss as “inverted erotic interests.” Paraphilias are characterized by persistent and recurrent sexual interests, urges, fantasies, or behaviors that involve atypical objects, activities, or situations. Although paraphilias themselves are not inherently pathological, they can develop into a paraphilic disorder if they cause harm, distress, or functional impairment to the individual or others. The *DSM‐5* (*Diagnostic and Statistical Manual of Mental Disorders*, fifth edition) lists eight specific paraphilias: pedophilia, exhibitionism, voyeurism, sexual sadism, sexual masochism, frotteurism, fetishism, and transvestic fetishism. Evaluating paraphilic disorders typically requires a thorough psychiatric assessment; however, there are currently no definitive emergency guidelines for assessing the severity of such behaviors in an acute setting. Various scales, such as the Paraphilias Scale and Freund Paraphilia Scales, can help quantify these behaviors [11, 12].

Management of paraphilic disorders is complex and generally involves both pharmacological and psychological approaches. Pharmacological options include selective serotonin reuptake inhibitors (SSRIs), anti‐androgens, and steroidal analogues, although their effectiveness can vary significantly. SSRIs and anti‐androgen medications, particularly GnRH agonists, have shown success in reducing sexual urges and behaviors in individuals at high risk of offending. However, many individuals with paraphilic disorders may be reluctant to seek treatment due to stigma or fear of legal repercussions. This reluctance can hinder the effectiveness of pharmacological interventions, which often require a holistic approach addressing both psychological and biological factors for optimal treatment outcomes [12, 13].

In our study, we report a case of self‐insertion of a sewing needle by an elderly man who presented to our emergency department with severe penile pain and itching. The needle was subsequently removed through cystoscopy after the patient was fully optimized for the procedure.

## 2 Case Presentation

### 2.1 Case History/Examination

A 65‐year‐old man with no known comorbidities presented to the emergency department with severe penile pain and itching that had begun the previous night. The pain was gradual in onset, described as pricking in nature, and it increased with movement while decreasing with rest. Upon physical examination, the penis was tender to the touch, but there was no swelling. During palpation, a long, hard object was detected in the bulbar urethra.

### 2.2 Imaging Investigation Findings

**FIGURE 1:**
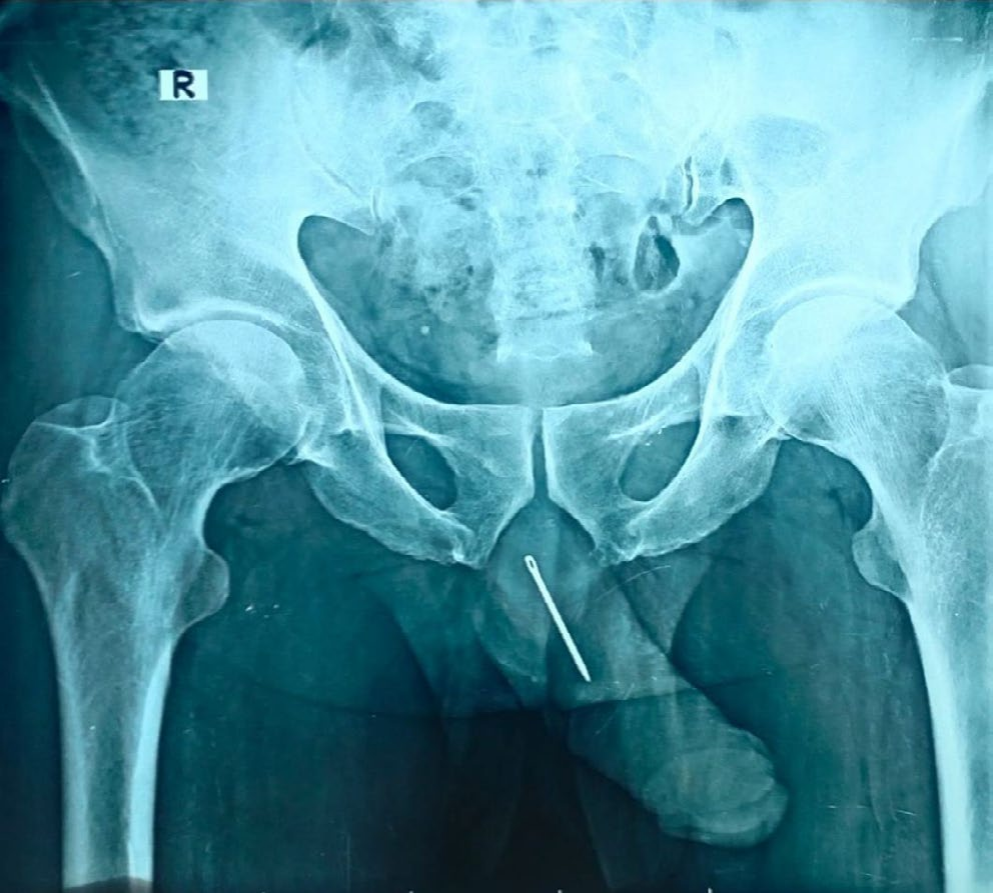
X‐ray pelvis anterior–posterior view showing a radiopaque foreign body (sewing needle) extending from the penile urethra to the root of penis (distal to proximal).

The patient's base line investigations were ordered. All base line investigations were normal. The complete urine report showed more than 200 red blood cells/μml and more than 80 white blood cells, whereas rest of the lab reports were un‐remarkable. His anterior–posterior view of the x‐ray pelvis depicted an elongated needle like radiopaque shadow of about 80 mm (8 cm) in length in area ranging from bulbar urethra to root of penis (Figure 1). The diagnosis of the foreign body inside the urethra was made.

### 2.3 Surgical Management

**FIGURE 2:**
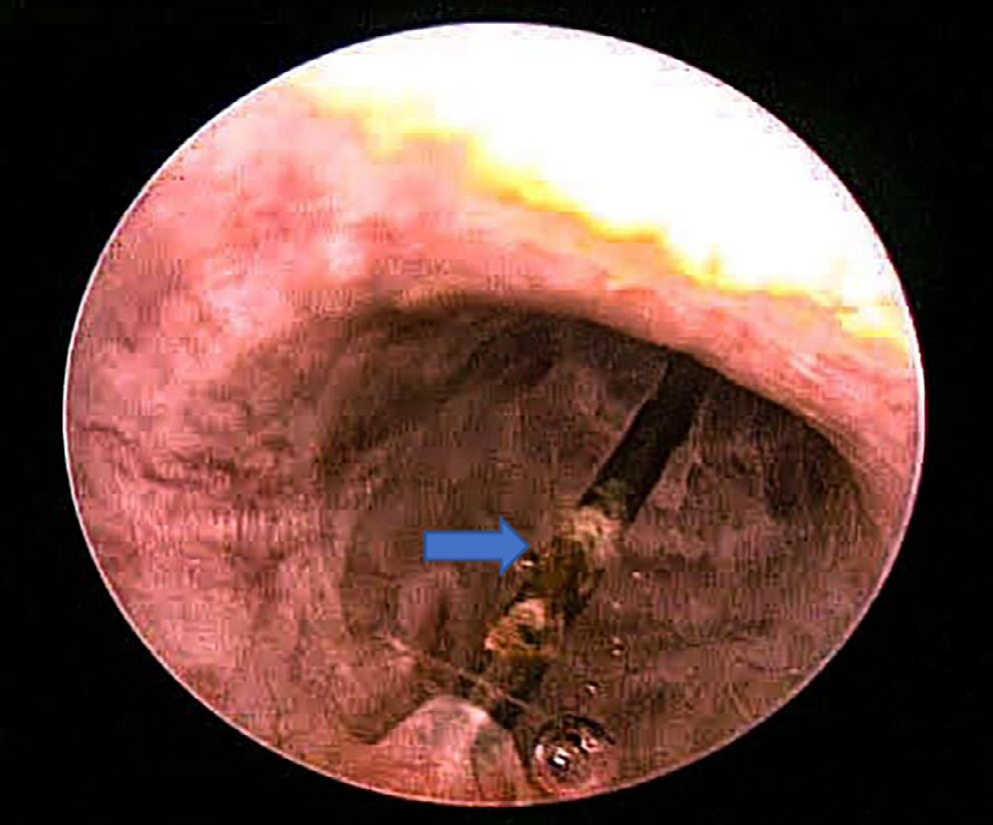
Cystoscopic removal of sewing needle through the urethra.

**FIGURE 3:**
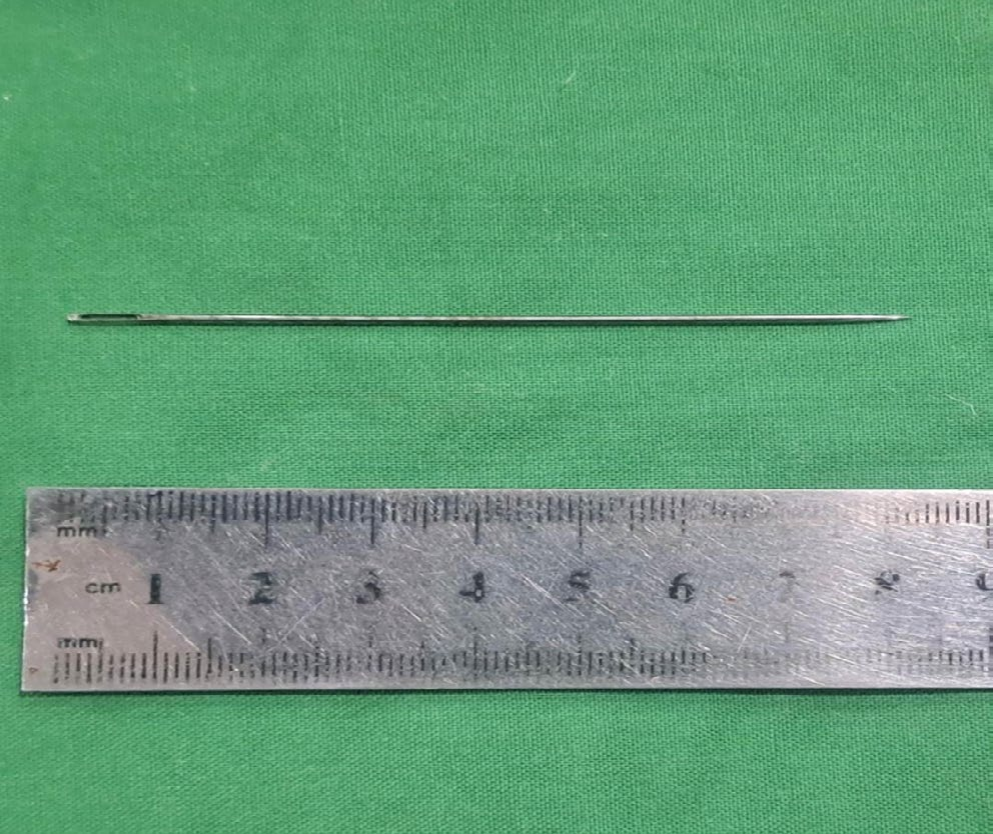
The length of needle retrieved was approximately 8 cm.

After complete evaluation of the patient by the anesthesia department, endoscopic removal was planned by cystoscopy under general anesthesia with a 22‐Fr sheath. The foreign body was localized in the bulbar urethra. The needle was grasped with the help of a grasper and removed gently through the urethral meatus (Figure 2). After removal of the sewing needle, its length was measured and documented (Figure 3). A 16‐Fr Foley catheter was passed into the patient's urethra and a urine bag was attached. Broad‐spectrum antibiotics were given. The catheter was removed after 1 week, and the patient's early mobilization was encouraged.

### 2.4 Ongoing Follow‐Up Plan

A comprehensive psychiatric evaluation was conducted by our Department of Psychiatry and Social Sciences. Initially, the patient denied any problematic behavior; however, he later acknowledged that he had engaged in self‐insertion practices for the past 10 years in response to extreme sexual urges. He reported that his first attempt occurred when he felt overwhelmed by these urges and experienced significant pleasure from the act. This behavior eventually became habitual. The situation escalated when he accidentally inserted a needle deeper into his urethra, leading him to seek emergency urological care. Prior to this incident, the patient had never undergone a psychiatric evaluation, as he had not encountered a situation as serious as the migration of the needle. A detailed history revealed that he had been living alone without a partner for over a decade. This prolonged period of isolation contributed to feelings of depression and psychosis, exacerbated by the absence of sexual intimacy. Our psychiatric team diagnosed him with fetishism, classified as a subtype of paraphilic disorder. As part of his treatment plan, the patient was prescribed a combination of pharmacotherapy and psychotherapy. Furthermore, he was encouraged to attend regular counseling sessions during follow‐up appointments to facilitate improvements in his behavior.

## 3 Discussion

Self‐insertion of objects into body orifices is observed in various psychiatric conditions, such as schizophrenia, bipolar disorder, manic episodes, and polyemobolokoilamania (a condition in which a person repetitively inserts various foreign bodies into body orifices to achieve sexual satisfaction) [14]. In the pediatric age group, children may insert objects out of curiosity about their bodies or for other unknown reasons. Additionally, foreign objects in the urethra are often found among specific groups, such as prisoners, who may seek to report persistent itching in the penile region due to urinary tract infections as a means to escape incarceration. Numerous cases of self‐insertion of foreign objects into the urethra have been reported in the literature worldwide [15, 16]. We noted significant variations in the types of objects inserted and the underlying motivations for this behavior, although most patients were men. These cases require thorough investigation before initiating any form of management. Frequently, an object that may be visible at the external urethral meatus can have its other end lodged in the urinary bladder. Randomly pulling such an object could result in urethral trauma. While x‐rays are typically the choice of investigation, ultrasound and computed tomography may also be necessary in certain cases.

Self‐inserted objects are managed based on their presentation. Objects that are visible externally are gently removed under local anesthesia with minor manipulation, whereas those that are displaced more proximally in the urethra may require more advanced urological care, such as cystourethroscopy or open surgical procedures.

In our study, we report a case of self‐insertion of a sewing needle by an elderly man who inserted the object to achieve sexual gratification. The sewing needle was successfully removed through cystoscopy. This study is comparable to several previously reported cases; for example, Untan et al. described an 18‐year‐old boy who presented with a self‐inserted, 15 cm long sewing needle that was removed via urethrocystoscopy [1]. In another study, a 14‐year‐old boy inserted a 9‐cm sewing needle into his urethra while engaging in auto‐erotic stimulation [2]. Additionally, Wang et al. documented a case of a 14‐year‐old boy who presented with a self‐inserted sewing needle measuring 46.9 mm, which was also removed through urethrocystoscopy [5].

**FIGURE 4:**
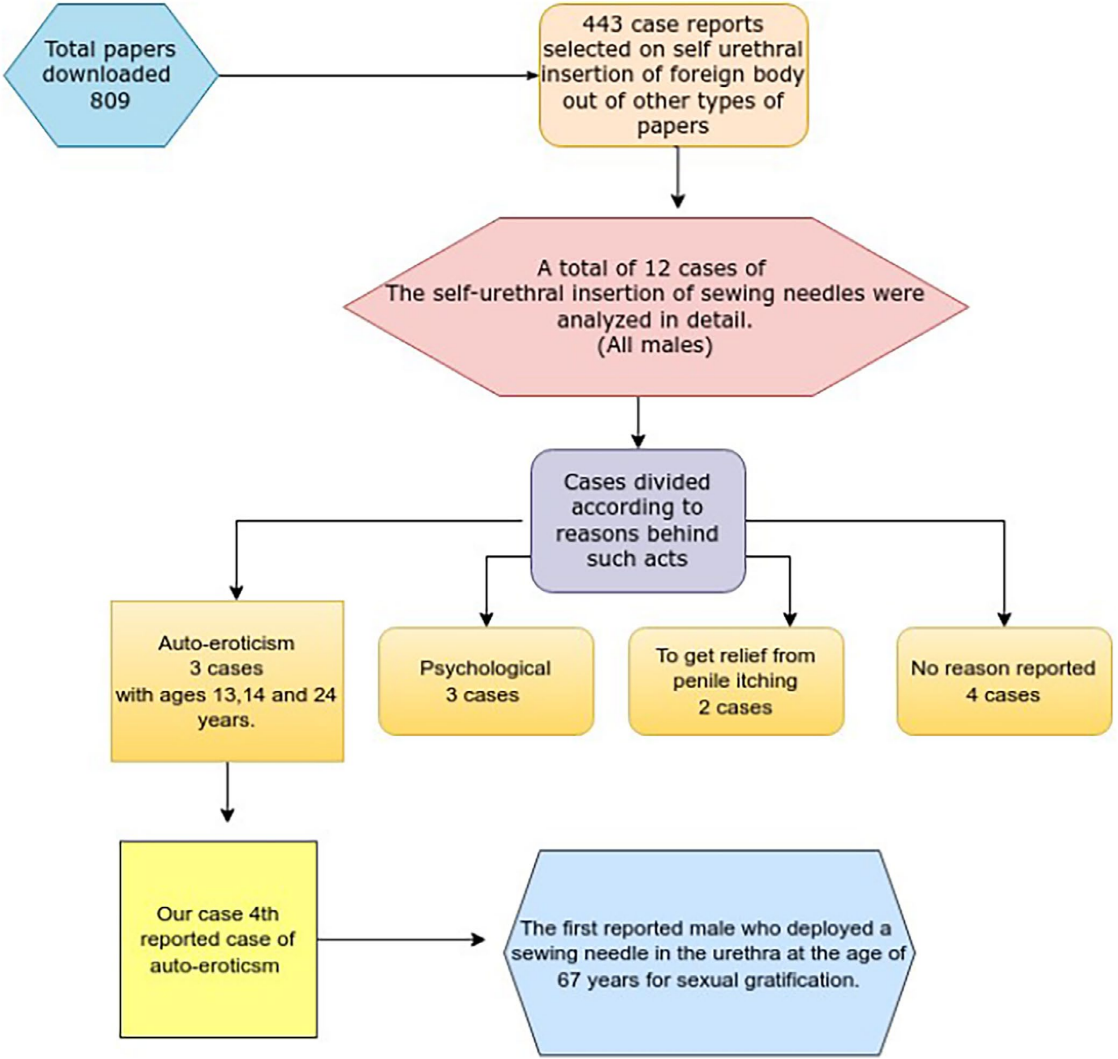
A flow diagram regarding review of the cases reported on self‐urethral insertion of sewing needle.

A basic review of PubMed indicates that approximately 809 papers on urethral foreign bodies have been published since 1872, including 66 reviews, 2 systematic reviews, and 443 case reports documenting urethral insertion of foreign bodies since 1961, with significant variation in the complexity of the objects (Figure 4). However, only 7 cases of urethral insertion of sewing needles are reported on PubMed, whereas 12 are found on Google Scholar, with 7 cases overlapping with those documented on PubMed. No cases have been identified in the Cochrane Library. Thus, we conducted a review of the total 12 cases of self‐urethral insertion of sewing needles documented globally to date, and we have tabulated our findings (Table 1). We found that all these cases involved males, with 10 cases managed through urethrocystoscopy, 1 case managed via laparoscopy, and 1 case addressed through gentle manipulation. Our case is unique, as it represents the fourth reported instance of auto‐eroticism involving the use of a sewing needle to achieve sexual pleasure and the first case globally of a male inserting a sewing needle into the urethra for sexual gratification at the age of 67 years. Many urological ailments can be masked by such foreign bodies when patients present to hospitals with complaints of lower urinary tract symptoms, as most individuals tend to conceal their acts of self‐insertion. Such cases require a high level of care for effective management [9, 10]. As highlighted by our literature review, there appears to be a diverse range of reasons behind the act of self‐insertion, making it challenging to screen patients who may be at risk for such behaviors. Although our literature review comprehensively addresses the reasons for self‐insertion, further psychiatric and research evaluation is necessary to fully understand the underlying causes. A thorough investigation into these reasons may help illuminate aspects of this issue, potentially reducing the incidence and morbidity associated with these acts.

**TABLE 1:** The comparative data of the previously reported cases of self‐urethral insertion of sewing needle.

Study	Year of case reported	Age of patient (years)	Gender	Length of sewing needle	Reason behind act of insertion	Presenting complaints	Method used for removal of object
Untan et al. [1]	2023	18	Male	1.5 cm	No reason reported	Urinary hesitancy, dysuria, epididmitis, orchitis	Urethro‐cystoscopy
Zaghbib et al. [2]	2019	14	Male	9 cm	Auto‐erotic stimulation	Dysuria	Urethro‐cystoscopy
Vahidi et al. [4]	2021	10	Male	8.7 cm	No reason reported	No significant symptom	Gentle Manipulation locally
Wang et al. [5]	2021	14	Male	5 cm	No reason reported	Tingling sensation of penis	Urethro‐cystoscopy
Ray et al. [6]	2015	12	Male	1.1 cm	Delusional disorder/psychological illness	Hematuria, dysuria	Urethro‐cystoscopy
Singh et al. [7]	2015	36	Male	7 needles of variable lengths	Polyembolokoilamania/psychological illness	Hard perineal swelling	Urethro‐cystoscopy
Bangash et al. [8]	2021	11	Male	2.5 cm	To get relief from penile itching	Poor urine stream and supra‐pubic pain	Urethro‐cystoscopy
Thakur et al. [9]	2022	24	Male	9 cm	Auto‐erotic stimulation	No symptom mentioned	Urethro‐cystoscopy
Lima et al. [10]	2019	11	Male	8 cm	Psychological illness	Penile pain	Urethro‐cystoscopy
Tahaoglu et al. [11]	2014	13	Male	Length not mentioned	Auto‐erotic stimulation	No specific symptoms	Urethro‐cystoscopy
Akdeniz et al. [16]	2020	11	Male	Length not mentioned	Due to penile itching	No specific symptoms	Urethro‐cystoscopy

## 4 Conclusion and Results

Numerous cases of self‐insertion of objects into the urethra have been reported in the literature globally, showcasing a wide range of structural variations. There are various reasons behind such acts, and a high level of expertise is required for management. Urologists should be aware of the possibility of foreign bodies in the urethra, as these can lead to serious complications if not diagnosed promptly. Patients presenting with lower urinary tract symptoms should undergo a detailed physical examination of the genitalia before any radiological investigations are conducted. The majority of foreign objects in the urethra necessitate cystoscopy for removal. Additionally, a psychiatric evaluation of these patients should be performed to gain a deeper understanding of the underlying reasons for such behaviors and to effectively address the root causes of their conditions.

## Author Contributions

**Rao Nouman Ali:** conceptualization, project administration, supervision, validation, visualization, writing – original draft, writing – review and editing. **Sohaib Irfan:** conceptualization, project administration, supervision, validation, visualization, writing – original draft, writing – review and editing. **Wajiha Irfan:** project administration, supervision, validation, visualization, writing – original draft. **Muhammad Asif Raza:** project administration, supervision, validation, visualization, writing – original draft. **Adeel Anwaar:** project administration, supervision, validation, visualization, writing – original draft. **Riyan Imtiaz Karamat:** data curation, visualization, writing – original draft, writing – review and editing. **Aymar Akilimali:** validation, visualization, writing – original draft, writing – review and editing. **Inam Ul Haq Arain:** validation, visualization, writing – original draft, writing – review and editing.

## Ethics Statement

This is a case report utilizing anonymized patient information and was therefore classified as exempt from review by the Institutional Review Board.

## Consent

A written informed consent was obtained from the patient based on the journal's policies.

## Conflicts of Interest

The authors declare no conflicts of interest.

## Data Availability

The authors have nothing to report.
